# Supplementary material for: Boosting reactivity of water-gas shift reaction by synergistic function over CeO2-x/CoO1-x/Co dual interfacial structures
Source: Nat Commun. 2023 Oct 27;14:6851. doi: 10.1038/s41467-023-42577-9 (PMC10611738; doi:10.1038/s41467-023-42577-9)
Supplement: Supplementary file 1 — Supplementary Information [file 41467_2023_42577_MOESM1_ESM.pdf]

Supplementary information for

Boosting reactivity of water-gas shift reaction by synergistic function over $CeO_{2-x}/CoO_{1-x}/Co$ dual interfacial structures

Xin-Pu Fu,[1,#] Cui-Ping Wu,[1,#] Wei-Wei Wang,[1] Zhao Jin,[1] Jin-Cheng Liu,[2,*] Chao Ma,[3,*] Chun-Jiang Jia[1,*]

[1]Key Laboratory for Colloid and Interface Chemistry, Key Laboratory of Special Aggregated Materials, School of Chemistry and Chemical Engineering, Shandong University, Jinan 250100, China.

[2]Center for Rare Earth and Inorganic Functional Materials, School of Materials Science and Engineering & National Institute for Advanced Materials, Nankai University, Tianjin 300350

[3]College of Materials Science and Engineering, Hunan University, Changsha, 410082, China.
[#]These authors contributed equally: Xin-Pu Fu, Cui-Ping Wu.

Table of Contents

Supplementary Methods

Supplementary Figures

Supplementary Tables

Supplementary Reference

Supplementary methods

Material. All materials were purchased from commercial vendors and used without further modifications or purification. Cobalt nitrate hydrate ($Co(NO_3)_2 \cdot 6H_2O$), cerium nitrate hydrate ($Ce(NO_3)_3 \cdot 6H_2O$), aluminum nitrate hydrate ($Al(NO_3)_3 \cdot 9H_2O$), and ethanol were purchased from Tianjin Kermal Chemical Reagent Factory.

Catalyst preparations

Preparation of CeO_2 nanoparticles. The preparation procedure referred to the research reported before.[1] Typically, 6 mmol of cetyltrimethylammonium bromide (CTAB) was dissolved in deoxidized water (200 mL), followed by introducing 10 mmol $Ce(NO_3)_3 \cdot 6H_2O$ inside. Subsequently, 0.2 mol·L^{-1} NaOH was dropwise added to the pre-mixed solution with vigorously stirring until the pH value reach 9. The suspension solution was further aged at 90 °C for another 3 h. After filtering and washing, the as-formed precipitate was dried at 110 °C in an oven for 12 h. Subsequently, the resultant powders were calcined at 400 °C for 5 h in the air (5 °C/min).

Characterizations

Scanning electron microscope (SEM). The SEM images were taken on a Zeiss SUPRA55 scanning microscope with an acceleration voltage of 5.0 kV.

X-ray photoelectron spectroscopy (XPS) analysis. Ten peaks were fit to the Ce 3*d* XPS spectrum and labeled according to Burroughs formalism, including five spin−orbit split doublets split by approximately 18.4 eV (v: $3d_{3/2}$; u: $3d_{5/2}$). For fitting purposes, the area intensity ratio $I(3d_{5/2})/I(3d_{3/2})$ was fixed to 1.5 for each doublet pair. The u′ and v′ peaks located at about 902.4 eV and 884.0 eV result from a $Ce3d^9 4f^1 O2p^6$ final state. The lowest binding energy states u^0 and v^0 located at 898.4 eV and 880 eV result from $Ce3d^9 4f^2 O2p^5$. An approximated calculation of the percent composition of Ce^{3+} species was obtained according to the following equation.

$$n(Ce^{3+})\% = \frac{A(u^0) + A(u')}{A(u^0) + A(u) + A(u') + A(u'') + A(u''')}$$

Transmission electron microscopy (TEM). The TEM and HRTEM images were taken from a FEI Tecnai F20 micorscope (200 kV) and Thermo scientific Themis Z microscope (300 kV) equipped with an imaging spherical-aberration corrector. A Thermo scientific Themis Z microscope, equipped with a probe-forming spherical-aberration corrector and Gatan image filter (Continuum 1077), was used to take the high-angle annular dark-field scanning transmission electron microscopy (HAADF-STEM)

images. The electron energy-loss spectroscopy (EELS) and X-ray energy-dispersive spectroscopy (EDS) were also recorded on the same equipment to obtain the elemental mappings of Co, Ce, and O atoms.

Surface area of catalysts. Builder SSA-4200 physisorption analyzer was used in measuring the surface area of catalysts. The catalysts were degassed at 200 °C for 6 h under vacuum before measurement. Surface area of each sample was calculated by the Brunauer-Emmett-Teller method.

X-ray diffraction (XRD). The ex-situ XRD patterns were obtained by a PANalytical X'pert3 powder diffractometer (40 kV, 40 mA, $\lambda_{Cu\text{-}K\alpha}$= 0.15418 nm) with an acquisition time of 8.5 min in the range of 10−90°. The *in-situ* XRD patterns were obtained from the same machine with an Anton Paar XRK-900 reaction chamber. Samples were loaded in a ceramic sample holder with a diameter of 10 mm and a depth of 1 mm. The *in-situ* reaction camber was heated from room temperature to 600 °C (interval: 100 °C) with a ramping rate of 30 °C/min under 5%H_2/Ar (30 mL/min). Two rounds of measurements, each lasting for 20 min, were carried out for each selected temperature. The second measurement round was collected and used to determine the structure of the catalysts.

Temperature Programmed Surface Reaction (TPSR). For both catalysts, the pretreated procedure was the same as for the catalytic tests above. After activation, samples were pre-hydroxylated with ~3%H_2O/Ar (30 mL/min) followed by Ar purging at 250 °C for another 30 min to purge the adsorbed H_2O. Subsequently, the gas was switched from Ar to 2%CO/Ar (30 mL/min) and then heated from room temperature to 400 °C (ramping rate: 5 °C/min). The outlet gases were analyzed by an online mass spectrometer (LC-D200M, TILON) with m/z = 28 (CO), 44 (CO_2), 2 (H_2).

Kinetics tests and calculation models

Apparent activation energy (E_a), Apparent kinetic orders, and kinetic isotopic effect (KIE). Appropriate amounts of catalysts diluted with SiO_2 were used in the kinetics experiments. The apparent activation energy and kinetic orders were performed on the same fixed-bed flow reactor mentioned above, with CO conversions in the range of 5%−15%. To measure the reactants (CO and H_2O) reaction orders of catalysts, the concentration of CO and H_2O were varied from 0.5%−10% with the reaction conversion remaining in the kinetic regime. The kinetic isotopic effect of catalysts was measured under a steady state at 250 °C, where feed gas alternatively changed from 2%CO/12%D_2O/N_2 to

$2\%CO/12\%H_2O/N_2$. The outlet gas was analyzed with the IR gas analyzer (Gasboard-3500, Wuhan Sifang Company, Wuhan, China).

Steady-state isotopic transient kinetic analysis (SSITKA). The SSITKA tests were measured in a self-conducted reactor, in which the inner diameter of the quartz reactor is 6 mm. A six-way valve was used to avoid the unnecessary signal perturb from gas switching. After the same activation pretreatment with catalytic tests, the catalysts were firstly subjected to $0.5\%^{12}CO/He$ with a space velocity of 120,000 $mL\cdot g_{cat}^{-1}\cdot h^{-1}$ in the absence of H_2O at 250 °C. After the signal reached a steady state, the gas flow was changed from $0.5\%^{12}CO/He$ to $0.5\%^{13}CO/He$. Similarly, the isotopic exchanges in the presence of H_2O were conducted with the same procedures, in which the feeding gas of $0.5\%^{12}CO/3\%H_2O/He$ was replaced by $0.5\%^{13}CO/3\%H_2O/Ar$. The reversibly adsorbed CO was derived from the *in-situ* $^{12}CO/He$ to $^{13}CO/Ar$ switches at 250 °C, which was representative of the total number of active sites (N_{total}). Subsequently, the adsorbed amount of CO (N_{CO}) under steady state was calculated by the *in-situ* switches between $^{12}CO/H_2O/He$ to $^{13}CO/H_2O/Ar$.
The corrected averaged residence time (τ) of CO was calculated based on the integrated area of the evolution curve detected by MS (^{12}CO or ^{13}CO; m/z = 28 or 29) excluding the corresponding integrated area of inert gas (He or Ar; m/z = 4 or 40).

$$\tau_{corrected} = \int_0^\infty F_{measured}(t)dt - \int_0^\infty F_{Ar}(t)dt$$

The number of surface adsorbed species (N*i*) was calculated as the following equation, where the *F* is the exit flow of CO:

$$N_i = \tau_{corrected} \times F$$

The coverage of CO was calculated based on the following equation:

$$\theta_{CO} = \frac{N_i}{N_{total}}$$

Computational model. CoO(100) surface was chosen in our work due to its high stability among the low-index ceria surfaces. CoO(100) was modeled by $p(4 \times 4)$ 4-layer supercells with the top two layers relaxed, and with a vacuum gap between slabs at ~15 Å. CeO_{2-x} is represented by a typical Ce_6O_{13} cluster reported in previous work.[2] $Co^0/CoO(100)$ model is represented by a Co_{10} metal cluster on CoO(100).

DFT Calculations. All DFT calculations were performed with the plane-wave basis sets of 400 eV cutoff kinetic energy to approximate the valence electron densities and projector-augmented wave method to account for the core–valence interaction,[3] as implemented in the Vienna Ab initio Simulation Package (VASP) code.[4,5] DFT + U method with U = 5 eV and 3.7 eV were used to describe the localized Ce 4f and Co 3d states, respectively.[6] The spin-polarized method with Perdew–Burke–Ernzerhof (PBE) flavor of generalized gradient approximation was employed.[7] Gamma centered Monkhorst−Pack (1×1×1) sampling was used for the Brillouin zone integration for all computational models due to the large cell. The convergence criteria for energy and force were set as 10^{-5} eV and 0.02 eV $Å^{-1}$, respectively. Transition states searched by climbing image nudged-elastic-band (CI-NEB) method with convergence criterion of 0.05 eV $Å^{-1}$.[8] Vibrational analyses were further performed to ensure the local minimum and transition states and vibrational frequencies.

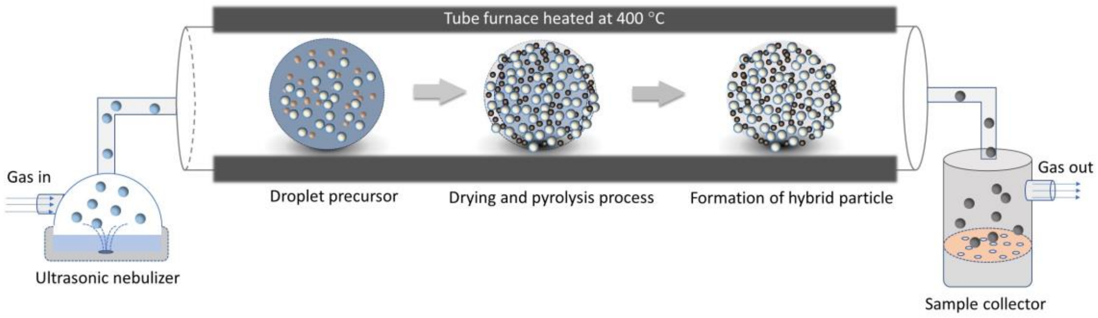

Supplementary Fig. 1. Schematically illustrating the preparation procedures of $CeCoO_x$ catalysts.

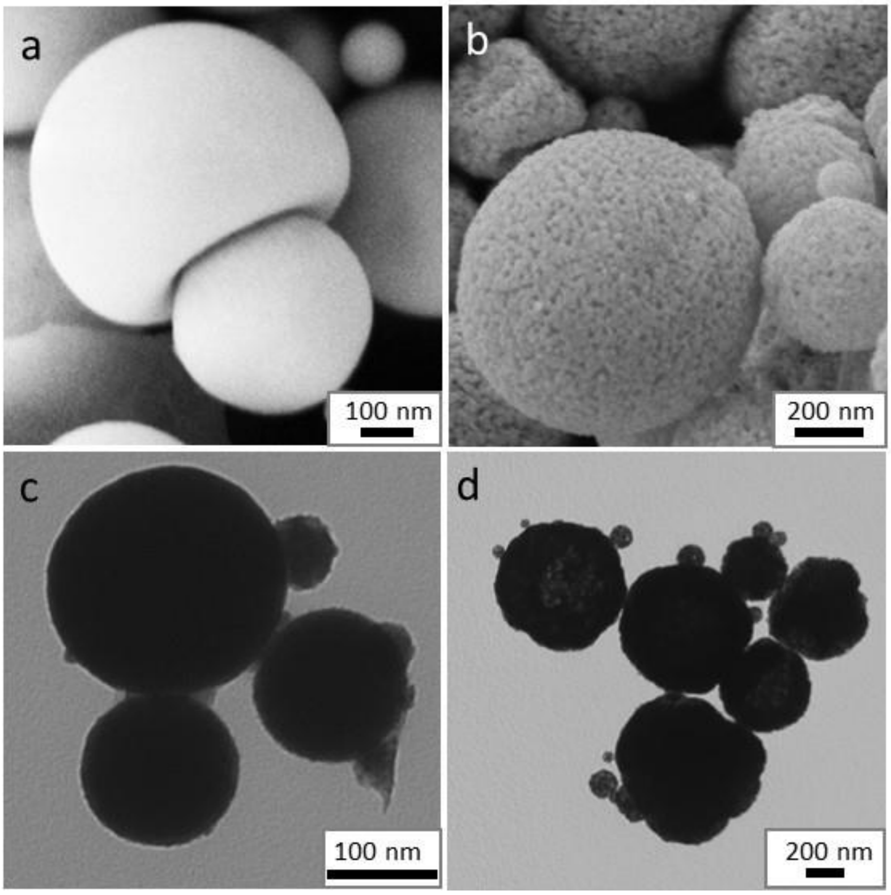

Supplementary Fig. 2. SEM (a and b) and TEM (c and d) images of fresh $CeCoO_x$ samples. The samples are $9Ce1CoO_x$ and Co_3O_4 respectively.

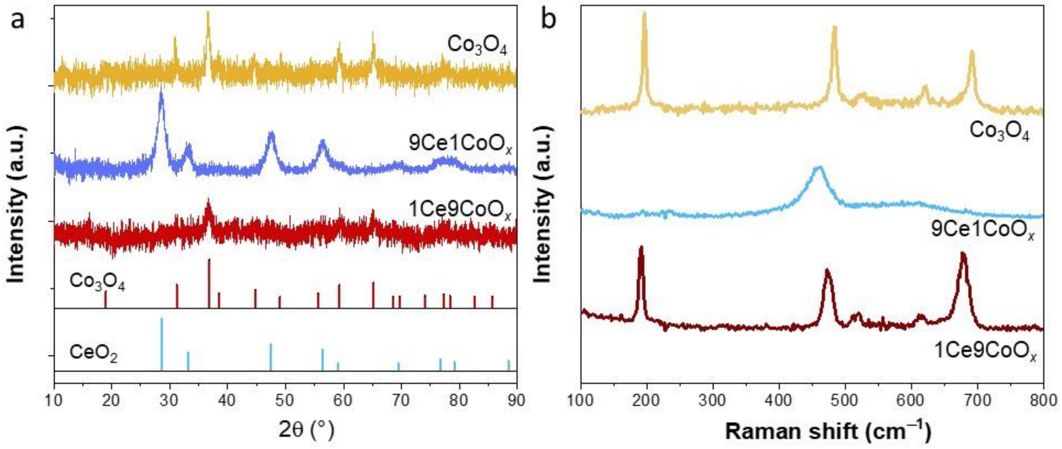

Supplementary Fig. 3. (a) XRD pattern and (b) Raman spectra of fresh $CeCoO_x$ catalysts. The diffracted peaks of crystallized CeO_2 are the merely detectable phase in the XRD pattern for $9Ce1CoO_x$.

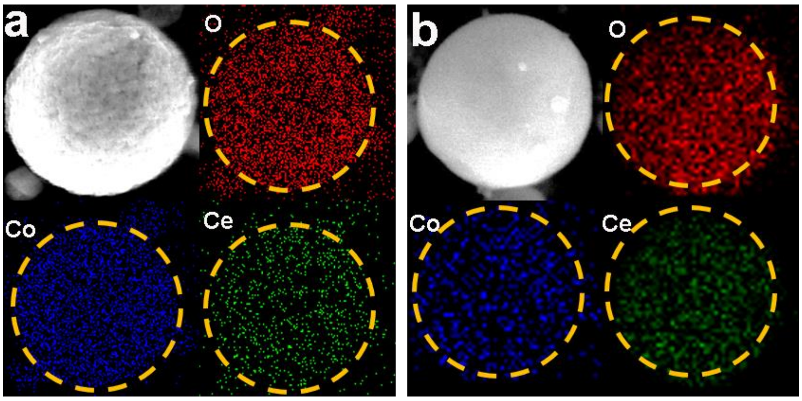

Supplementary Fig. 4. SEM image and elemental mapping of (a) fresh 1Ce9CoO$_x$ and (b) 9Ce1CoO$_x$ catalysts.

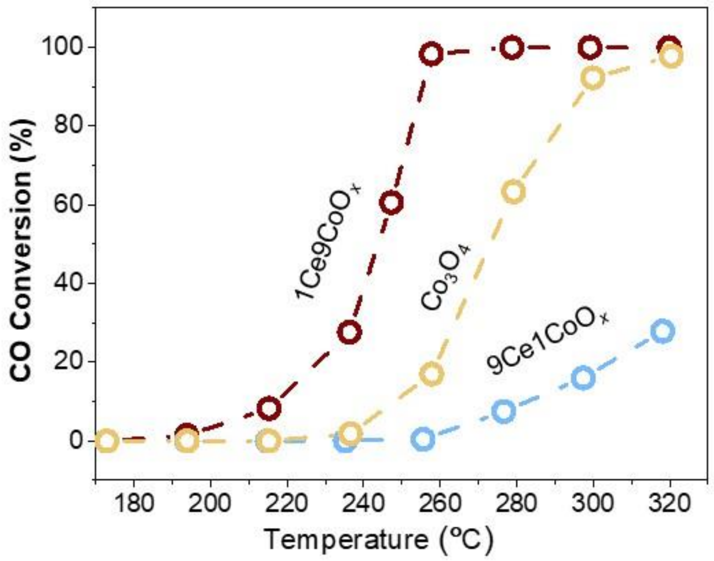

Supplementary Fig. 5. CO conversion on CeCoO$_x$ and Co_3O_4 catalysts at various temperatures. Reaction condition: 2%CO/10%H_2O/Ar; GHSV = 168,000 mL·g^{-1}·h^{-1}.

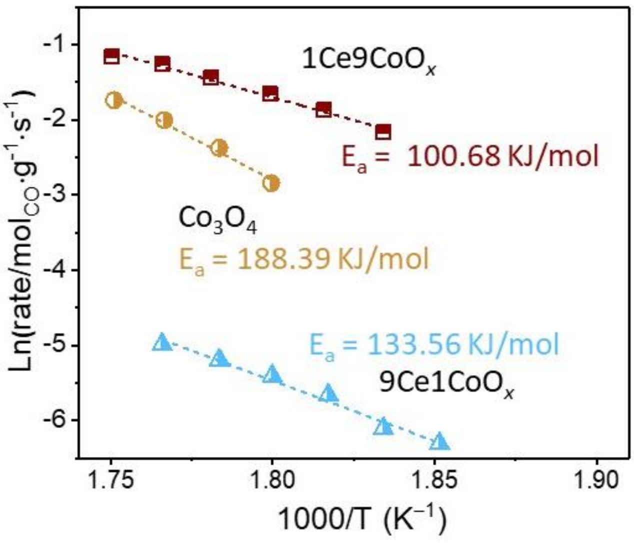

Supplementary Fig. 6. The apparent activation energy of the 1Ce9CoO$_x$, Co_3O_4 and 9Ce1CoO$_x$ catalysts.

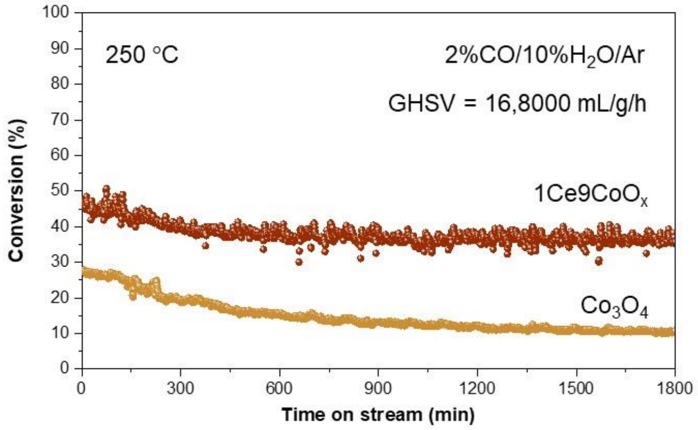

Supplementary Fig. 7. Long-term WGS reaction test over the 1Ce9CoO$_x$ and Co_3O_4 catalysts at 250 °C. The other Reaction condition: 2%CO/10%H_2O/N_2, GHSV = 168,000 mL/g$_{cat}$/h.

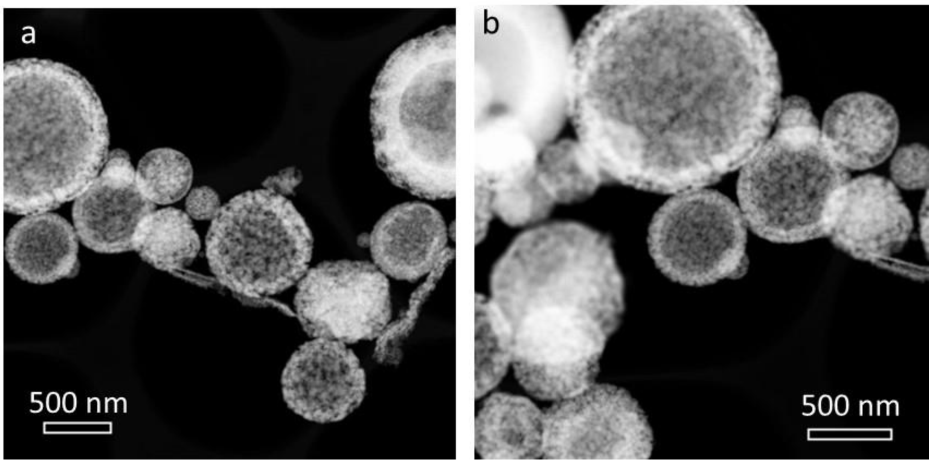

Supplementary Fig. 8. (a and b) HAADF-STEM image collected for the spent 1Ce9CoO$_x$ catalyst. The morphology of the 1Ce9CoO$_x$ catalyst remains spherical structure after the WGS reaction, indicating the good stability of nano-hybrid with the presence of CeO$_x$ species.

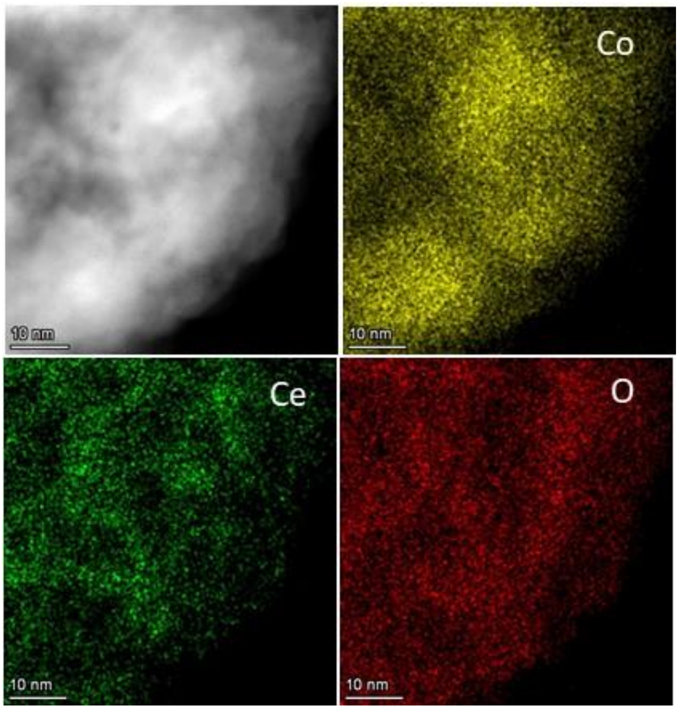

Supplementary Fig. 9. X-ray EDS elemental mapping of Co, Ce and Co for the selected region of the spent 1Ce9CoO$_x$ catalysts. The CeO_2 particles were located in the interstitial region of Co species. This indicated that the length of the Co(O$_x$)/CeO_2 interface was delimited by the perimeter of CeO_2 nanoparticles, which therefore induced relatively excellent stability of 1Ce9CoO$_x$ under WGS reaction condition owing to the more stable nature of CeO_2 than Co species.

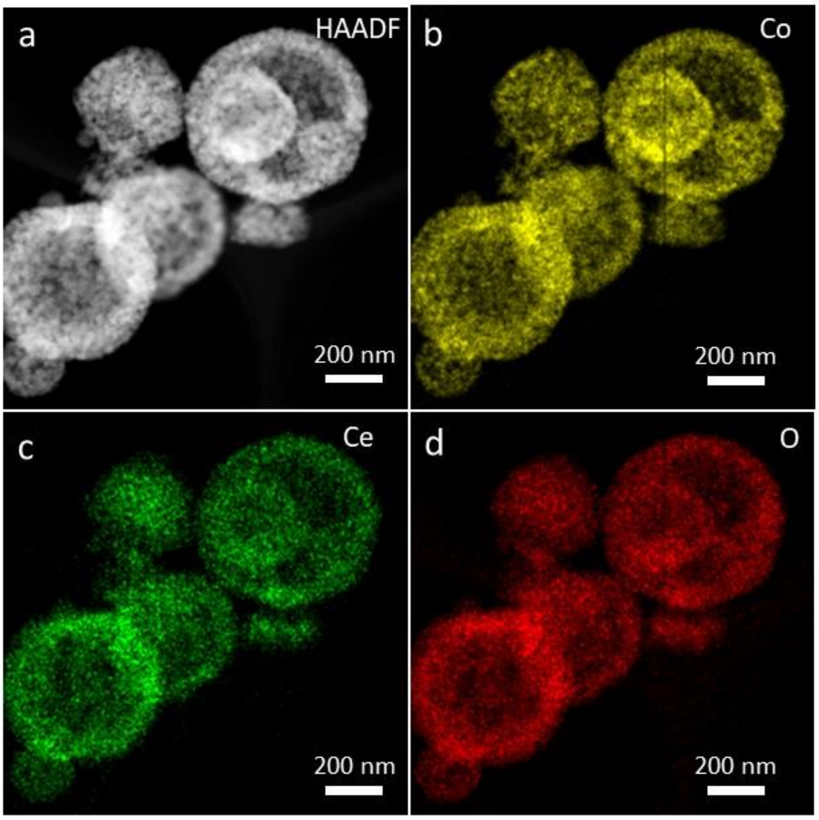

Supplementary Fig. 10. (a) HAADF-STEM image and (b-d) elemental mapping (Co, Ce and O) of 1Ce9CoO$_x$ collected after long-term WGS reaction. The Co and Ce elements are homogenously dispersed within the integrated sphere morphology, indicating the obbligato role of CeO_2 islets in stabilizing Co species.

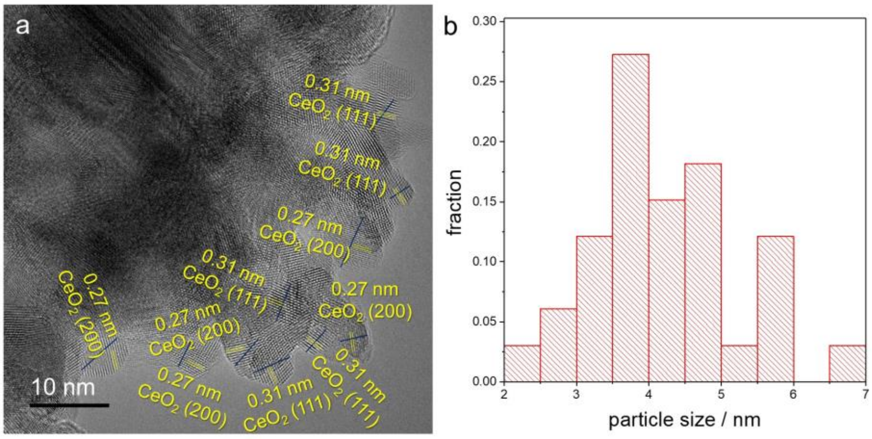

Supplementary Fig. 11. Statistic distribution of CeO_2 particle size.

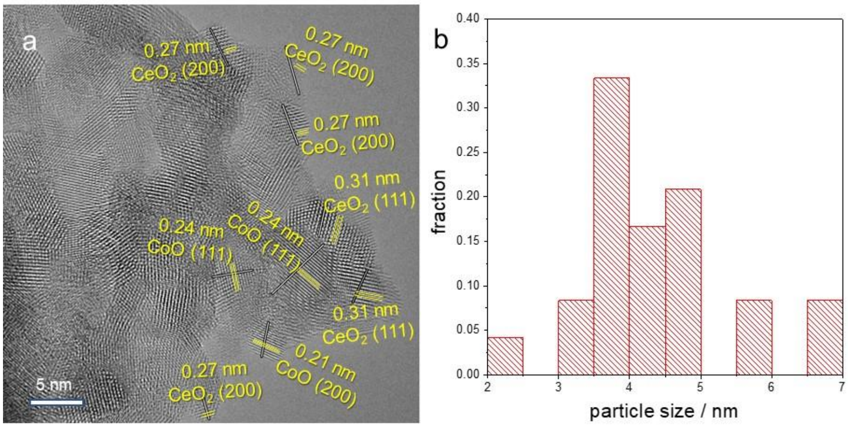

Supplementary Fig. 12. Statistic distribution of CoO and CeO_2 particle size.

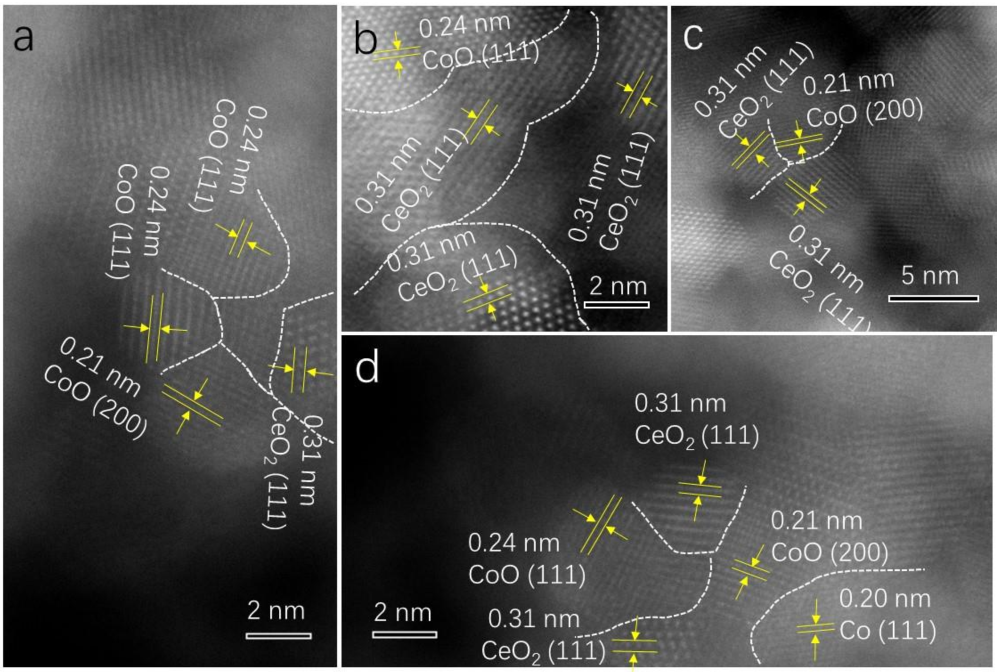

Supplementary Fig. 13. (a-d) Atomic-resolution HAADF-STEM images of the selected area of $1Ce9CoO_x$ after transient WGS reaction to determine the microstructure of spent catalysts. The CoO nanoparticles are surrounded by a few small CeO_2 nanoparticles with a diameter of about 2−5 nm.

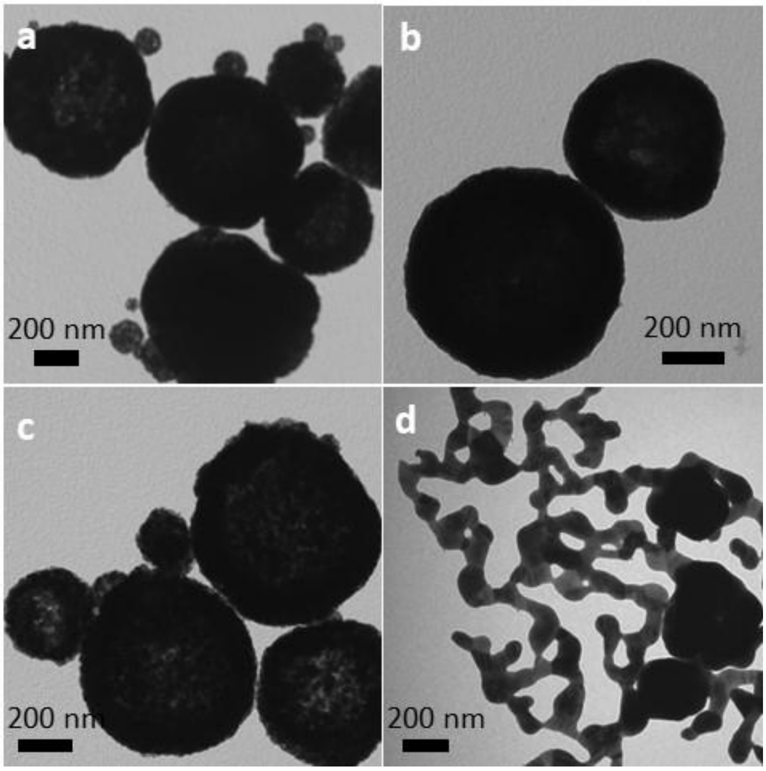

Supplementary Fig. 14. TEM images of (a) fresh and (b) spent 1Ce9CoO catalysts; (c) fresh and (d) spent Co_3O_4 catalyst. The nanoparticles in Co_3O_4 were seriously sintered after the WGS reaction, resulting in a dynamic drop in the CO conversion during the long-term test.

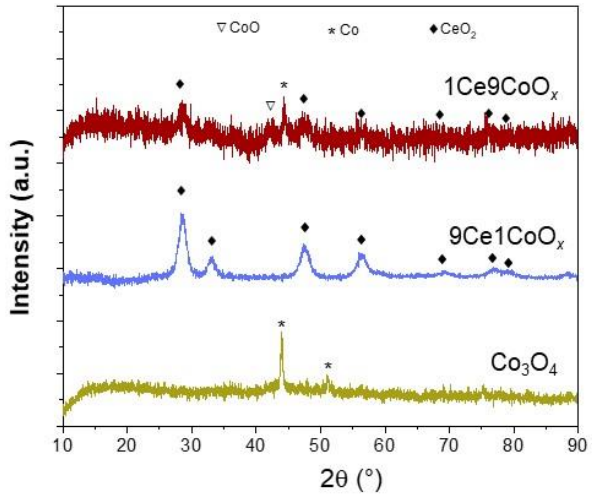

Supplementary Fig. 15. XRD pattern and of spent CeCoO$_x$ and Co_3O_4 catalysts. As confirmed by the XRD pattern collected after the WGS reaction, the spent Co_3O_4 catalyst dominantly comprised metallic Co^0 species without the formation of detectable CoO, indicating the presence of CeO_2 might be essential for stabilizing the CoO phase under WGS reaction condition.

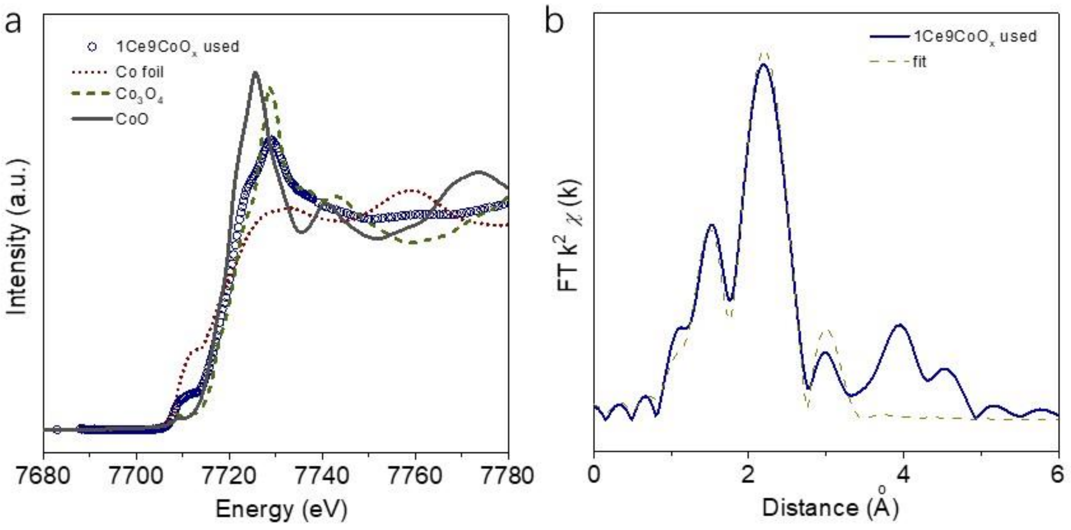

Supplementary Fig. 16. (a) XANES and (b) EXAFS results of spent 1Ce9CoO_x catalysts collected for the second time, where the catalyst was protected by N_2 before experiments. As shown in Supplementary Fig. 16, both Co-Co bond (d = 0.250 ± 0.001 nm, CN = 6.9 ± 0.4) and Co-O bond (d = 0.190 ± 0.001 nm, CN = 3.4 ± 0.2) could be observed over the EXAFS profile, indicating that the co-presence CoO_x and metallic Co after the WGS reaction condition.

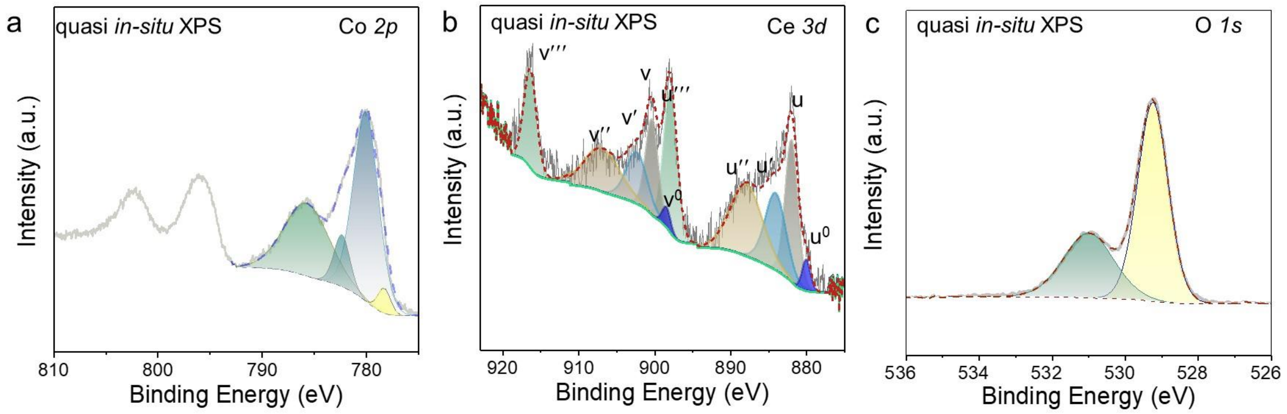

Supplementary Fig. 17. Quasi *in-situ* XPS results of 1Ce9CoO$_x$: (a) Co *2p*, (b) Ce 3*d* and (c) O 1*s* XPS spectra. The XPS spectra were collected after *in-situ* pretreated by 5%H_2/Ar at 400 °C for 1 h and *in-situ* WGS reaction at 250 °C for 1 h (2%CO/~3%H_2O/Ar). Apart from the B.E. signal identified to the lattice O atoms, the O *1s* XPS profile centered at ~531.2 eV ascribed to the O atoms close to O_v was observed,[9,10] indicating the boosted presence of O vacancies after *in-situ* WGS reaction as compared with fresh catalysts (Fig. 2e).

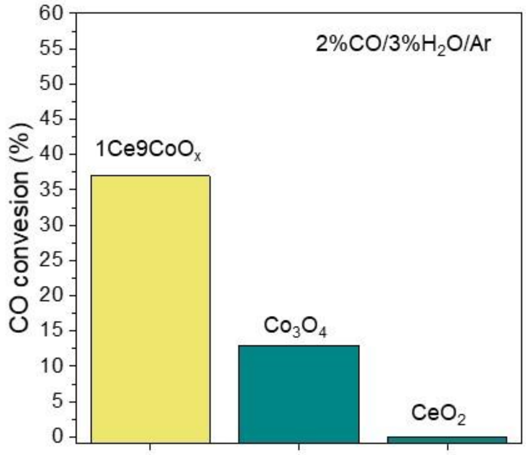

Supplementary Fig. 18. The comparison of the CO conversions for the WGS reaction performed at 260 °C over the 1Ce9CoO$_x$, Co_3O_4 and CeO_2 catalysts, where the atmospheres were comparable with the *in-situ* Raman and (quasi) *in-situ* XPS experiments (2%CO/3%H_2O/Ar).

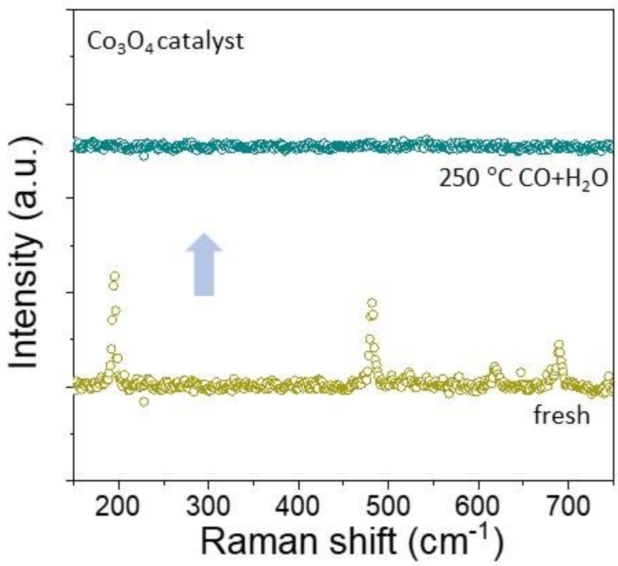

Supplementary Fig. 19. *In-situ* Raman spectra collected under WGS reaction condition at 250 °C for the Co_3O_4 catalyst. The absence of Raman signal for the Co_3O_4 catalyst implied that the Co species dominantly present as Co^0 under WGS reaction condition.

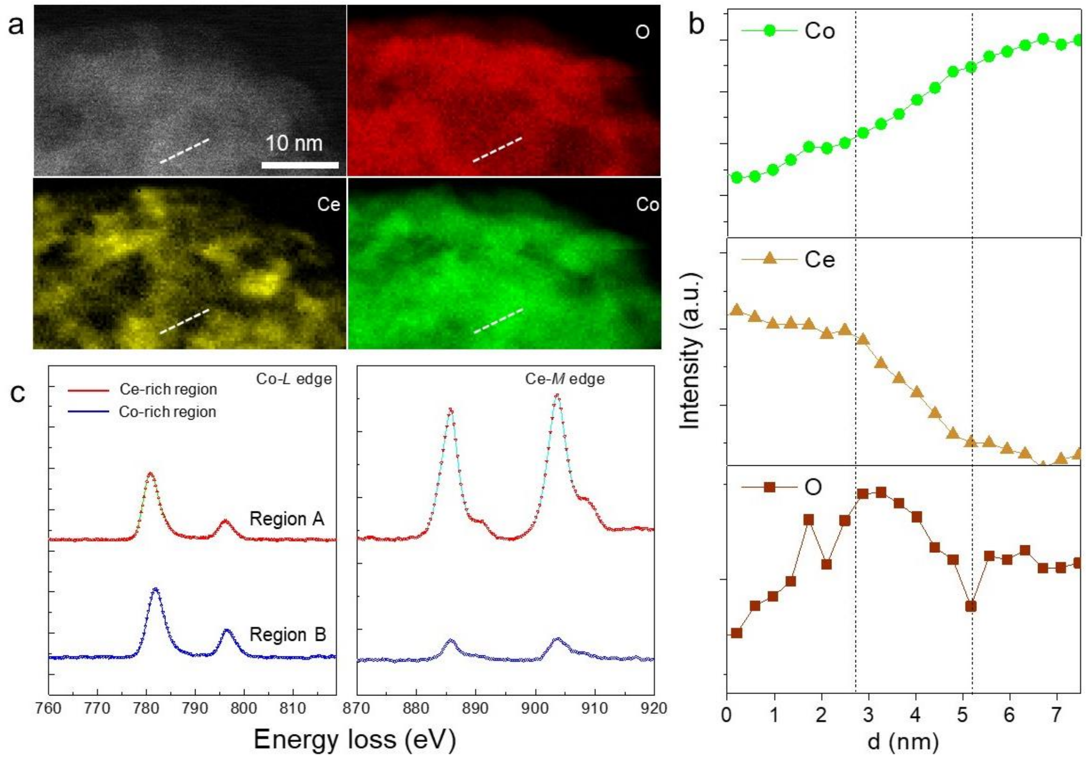

Supplementary Fig. 20. (a) STEM-EELS mapping results, (b) EELS intensity profiles of Co-*L*, Ce-*M*, and O-*K* edges following the selected dotted line in Supplementary Fig. 20a and (c) Co-*L* and Ce-*M* edge EELS spectra collected within various regions over the spent 1Ce9CoO_x catalyst.

The electron energy-loss spectrum (EELS) is sensitive to the chemical environment of the selected region, which thus could be used to probe the oxidation state. As shown in Supplementary Fig. 20, the Ce species were normally distributed among the space interval of Co species with a diameter in the range of 3–5 nm, which was consistent with the statistic size for CeO_2 based on the HRTEM image. The gradient evolution of Co and Ce intensity (Supplementary Fig. 20b) was reversed along the selected dotted line in Supplementary Fig. 20a, indicating the presence Co-CeO_2 interface within in the observed region.

To further identify the interfacial Co-CeO_2 structure, we collected the Co-*L* and Ce-*M* edges spectra within two typical regions: Ce-rich region (R-A) and Co-rich region (R-B). The creation of oxygen vacancies was proved by the energy shift within the Co-$L_{2,3}$ edge spectra when the Co atoms were adjacent to the CeO_2 species.[11,12] The intensity ratio of L_3/L_2 is determined by the occupation state of 3*d*-states, which thus could monitor the electronic change induced by the creation of oxygen vacancies within the interface region. The calculated L_3/L_2 ratio for Ce *L*-edge collected in R-A was ca. 4.8, while

the corresponding values calculated for R-B decreased to 3.2. The decreased L_3/L_2 ratio suggested the presence of CoO_{1-x} species at the interface.

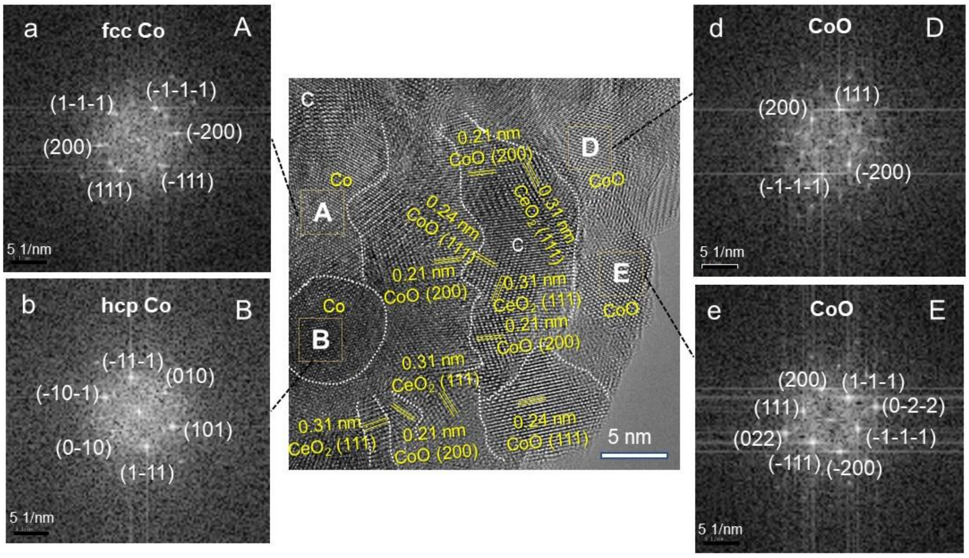

Supplementary Fig. 21. (c) HRTEM image for the selected area of 1Ce9CoO$_x$ after transient WGS reaction, where the interfacial regions were marked with dotted line; (a, b, d, e) the corresponding FFT images obtained from the region A, B, D and E, as illustrated in Supplementary Fig. 21c.

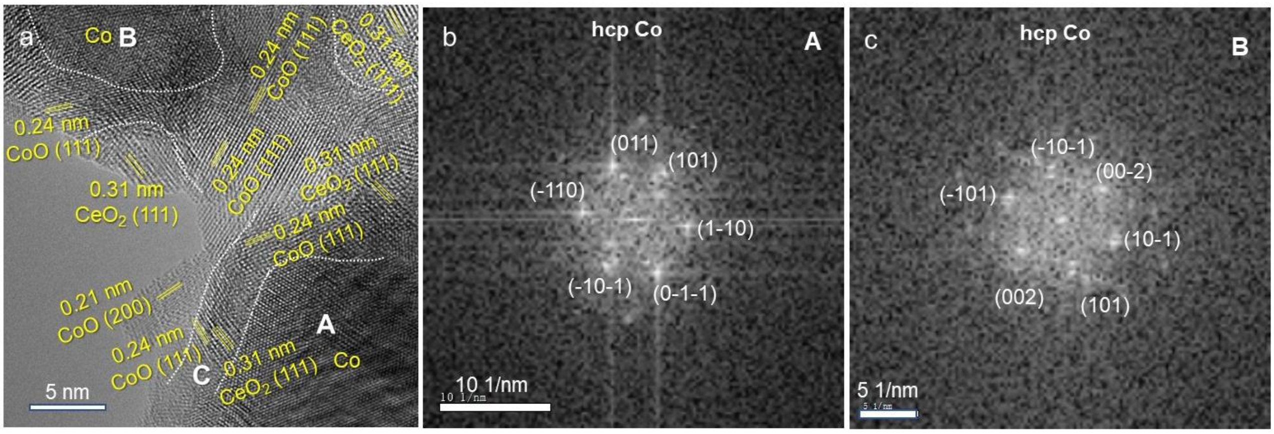

Supplementary Fig. 22. (a) HRTEM image for the selected area of 1Ce9CoO$_x$ after transient WGS reaction, where the interfacial regions were marked with dotted line; (b) and (c) the corresponding FFT image obtained from the region A and B as illustrated in Supplementary Fig. 22a.

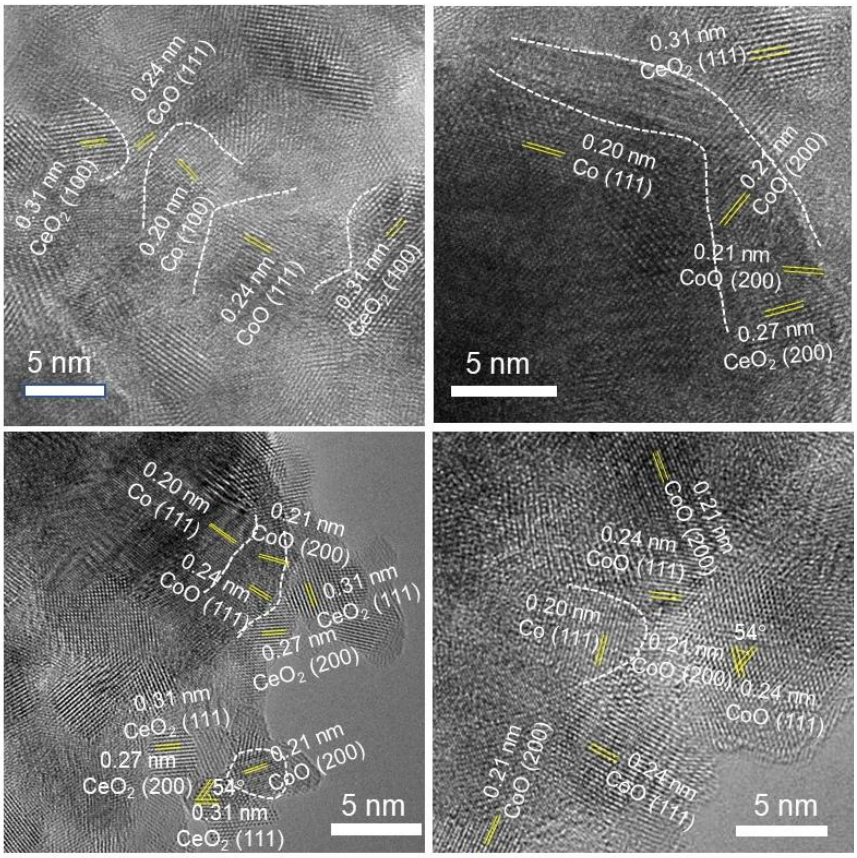

Supplementary Fig. 23. HRTEM images of spent 1Ce9CoO$_x$ catalyst, where the interfacial regions were marked with dotted line.

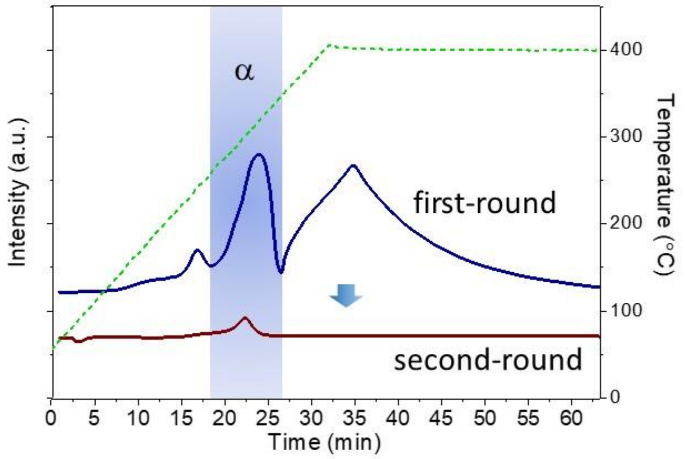

Supplementary Fig. 24. Consecutive H_2-TPR profile collected without exposing to air for the 1Ce9CoO$_x$ catalysts. Prior to conducting the first-round H_2-TPR experiment from 30-400 °C, the 1Ce9CoO$_x$ was pretreated by the air at 300 °C for 30 min. Subsequently, the second-round H_2-TPR profile was collected after the sample cooling down in 5%H_2/Ar without exposing to any O-containing gas. The regeneration of α peak centered at ca. 300 °C implied that the reduced Co species can be partially oxidized by the CeO_2.

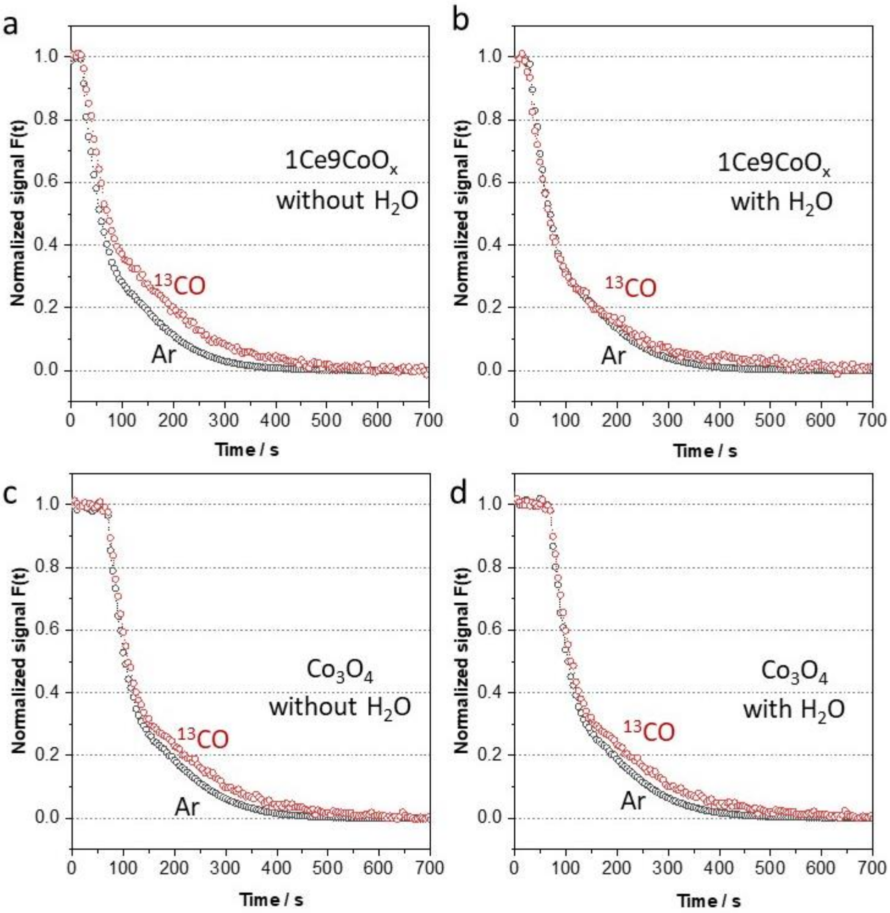

Supplementary Fig. 25. The steady-state isotopic transients from (a) He/^{12}CO to Ar/^{13}CO and (b) He/^{12}CO/H_2O to Ar/^{13}CO/H_2O at 250 °C for 1Ce9CoO$_x$ catalyst. The steady-state isotopic transients were performed by switching from (c) He/^{12}CO to Ar/^{13}CO and (d) He/^{12}CO/H_2O to Ar/^{13}CO/H_2O at 250 °C for the Co_3O_4 catalyst. All the tests were conducted after *in-situ* pretreatment under 5%H_2/Ar at 400 °C for 1 h.

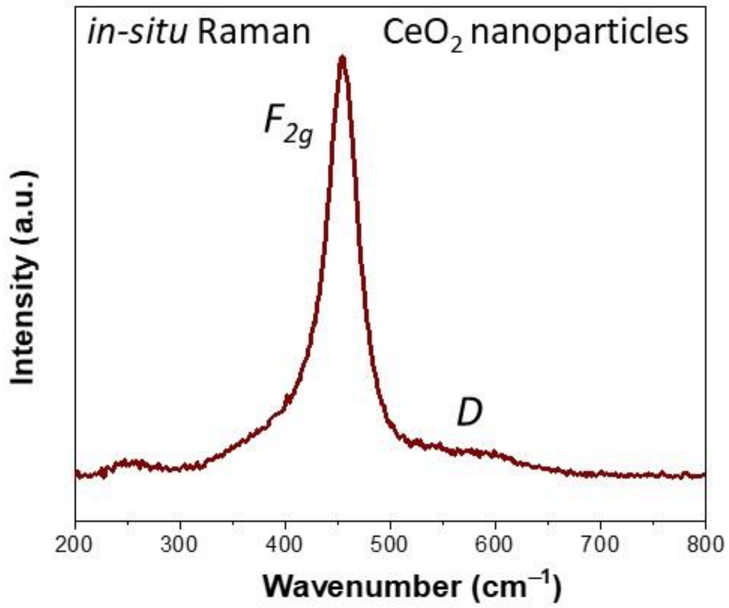

Supplementary Fig. 26. *In-situ* Raman spectrum collected under 5%H_2/Ar at 400 °C for CeO_2 nanoparticles.

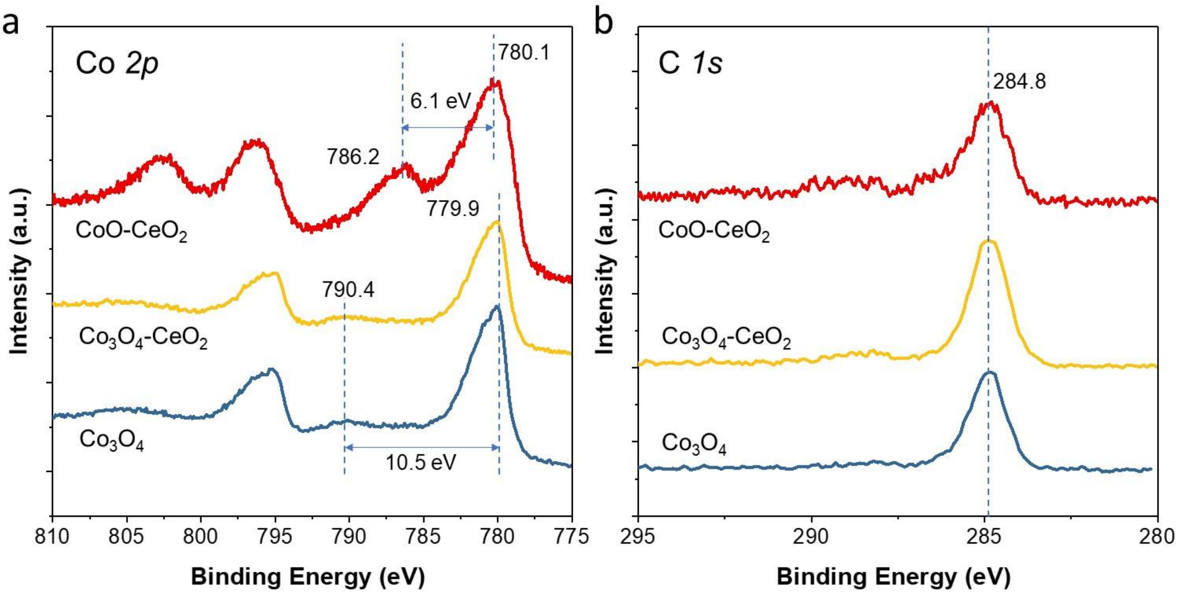

Supplementary Fig. 27. XPS spectra of three kinds of Co-based catalysts: Co_3O_4, Co_3O_4-CeO_2 and CoO-CeO_2. (a) Co *2p* XPS spectra and (b) C *1s* XPS spectra.

The structure of reference catalysts was determined by XPS characterizations. One strong signal at 779.9 eV coupling with a weak satellite peak at 790.4 eV as shown in Co *2p* XPS spectra for Co_3O_4/CeO_2 confirmed that the Co_3O_4 was the initial phase (Supplementary Fig. 27), which was the same with bare Co_3O_4 catalyst. The binding energy of Co 2p XPS for the CoO/CeO_2 catalyst was centered at 780.1 eV, which could be ascribed to the dominant presence of Co^{2+}. The characteristic distance of 6.1 eV between Co $2p_{3/2}$ and the strong satellite peak indicates the dominant presence of CoO.

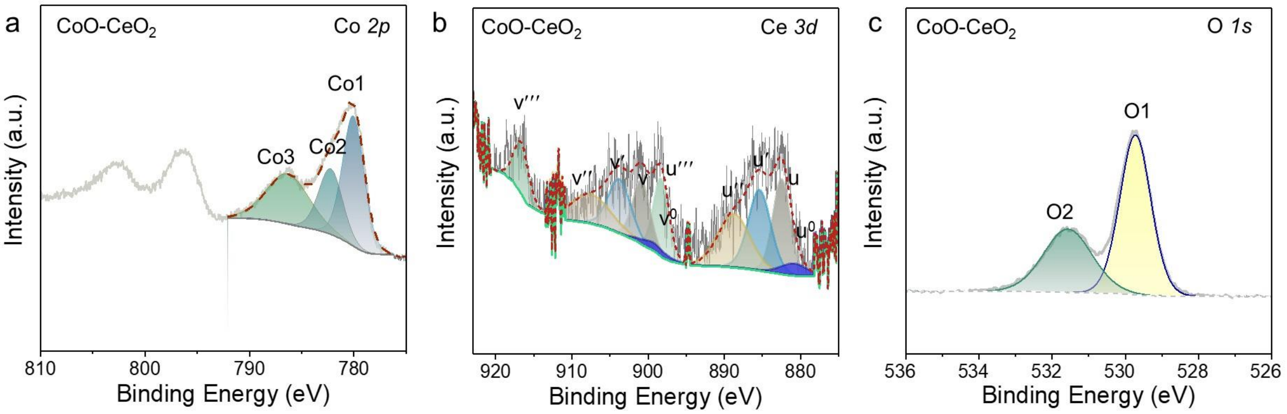

Supplementary Fig. 28. XPS results of $CoO-CeO_2$: (a) Co 2p, (b) Ce 3d and (c) O 1s XPS spectra.

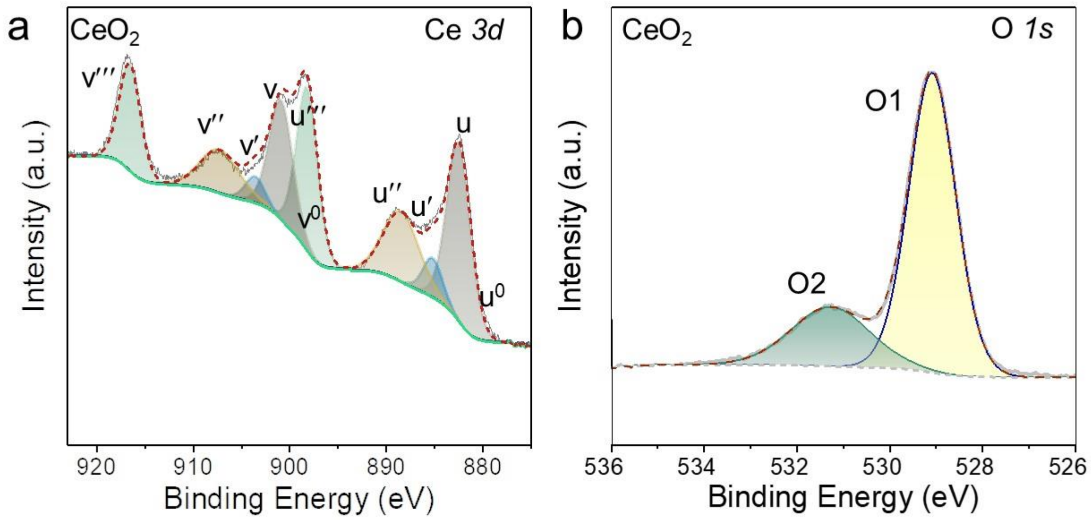

Supplementary Fig. 29. XPS results of bare CeO_2: (a) Ce *3d* and (b) O *1s* XPS spectra.

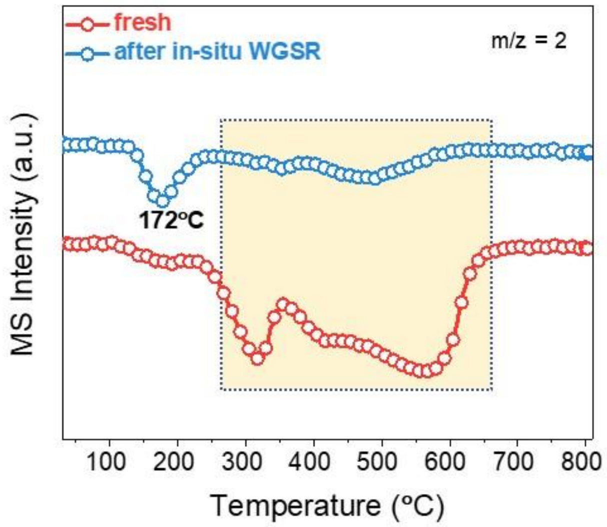

Supplementary Fig. 30. H_2-TPR profiles for the fresh 1Ce9CoO_x (red) and spent 1Ce9CoO_x catalysts which pretreated by *in-situ* WGS reaction (blue).

To further prove the presence of Co-CeO_2 interaction after WGS reaction, the reduction behavior of Co-based species determined by the H_2-TPR test could give a strong hint about the phase information and chemical interaction between heterogenous species. Therefore, we collected the H_2-TPR profile for the 1Ce9CoO_x catalysts after the *in-situ* WGS reaction (250 C, 2%CO/3%H_2O/Ar), which can rule out the interference from oxidation during other ex-situ characterizations. As shown in Supplementary Fig. 30, the first round of H_2-TPR result demonstrated a typical evolution for the multi-step reduction of Co_3O_4 species. After *in-situ* WGS reaction treatment, the presence of the reduction peak at 400–600 °C implied that the interfacial CoO species was maintained during the WGS reaction. These H_2-TPR results evidenced the presence of the CoO_x species even after H_2-pretreatment and subsequent WGS reaction; on the other hand, the dominant presence of active O species was in line with the proposed redox mechanism upon the surface of the catalyst.

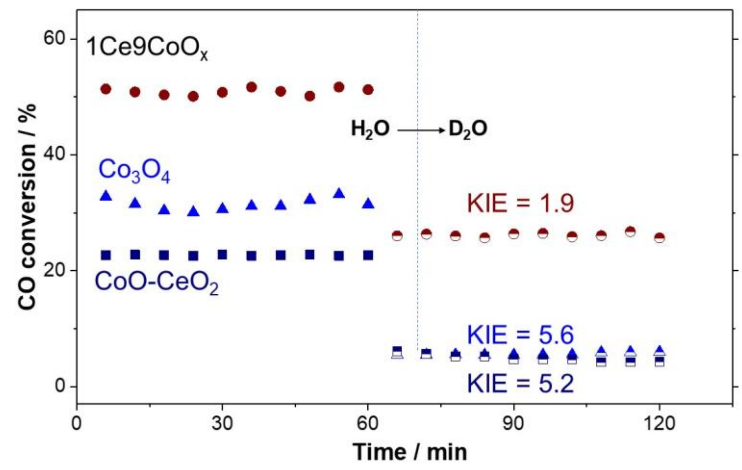

Supplementary Fig. 31. The H/D kinetic isotopic effect of 1Ce9CoO$_x$, CoO-CeO$_2$ and Co_3O_4 catalysts, where the WGS reaction tests were conducted at 250 °C with the atmosphere changed from 2%CO/10%H_2O/Ar to 2%CO/10%D_2O/Ar.

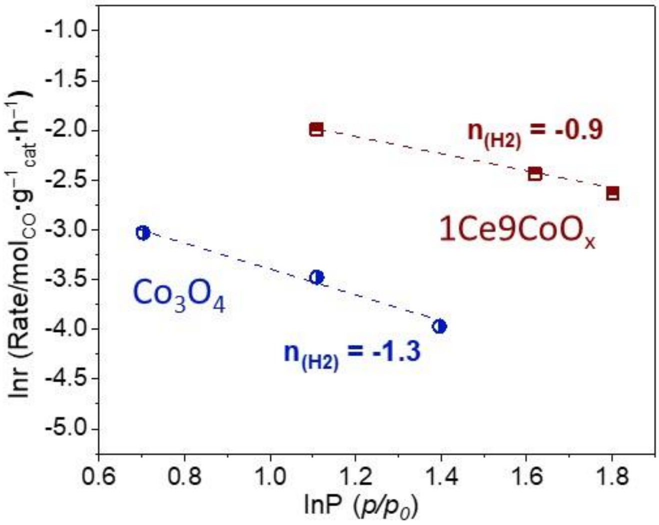

Supplementary Fig. 32. Apparent reaction order of H_2 molecules for the 1Ce9CoO$_x$ and Co_3O_4 catalysts. The reverse-first order of H_2 for both catalysts indicated that the H_2 formation or desorption step is relatively sensitive to the coverage of surface H species. In addition, the value of −0.9 and −1.3 for catalysts might be owing to that the adsorbed H species onto Co^0 sites would compete with CO adsorption; or the H transfer step will compete with H_2O dissociation.

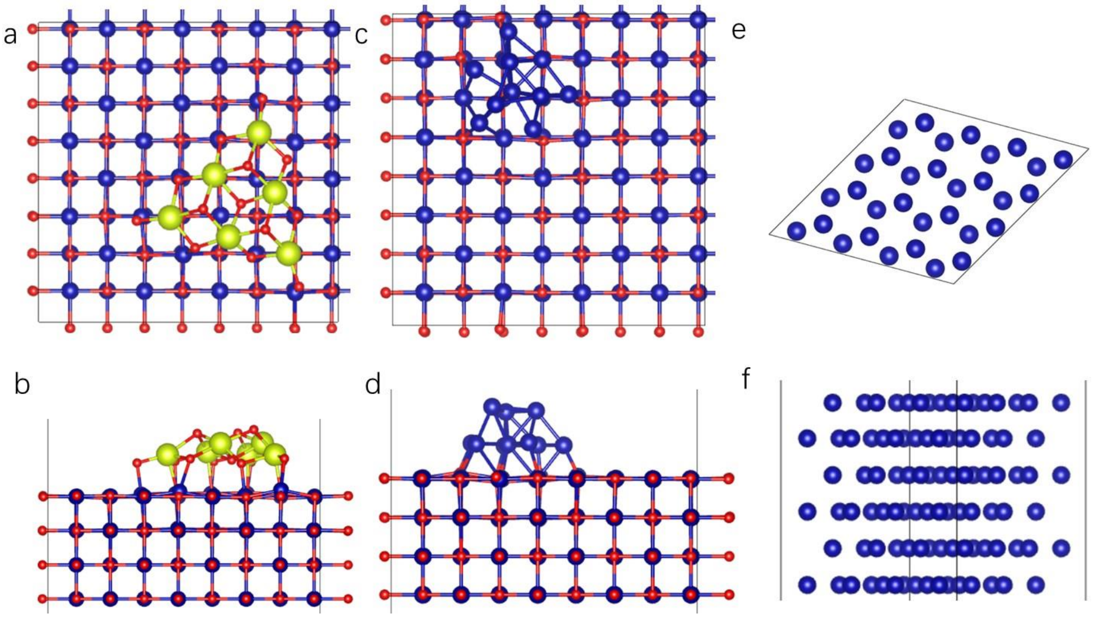

Supplementary Fig. 33. Top and side view of three catalyst models based on the experimental results, Co(0001), CeO_{2-x}/CoO(111) and Co_8/CoO(111); Co, Ce, O atoms are shown in purple, yellow and red, respectively.

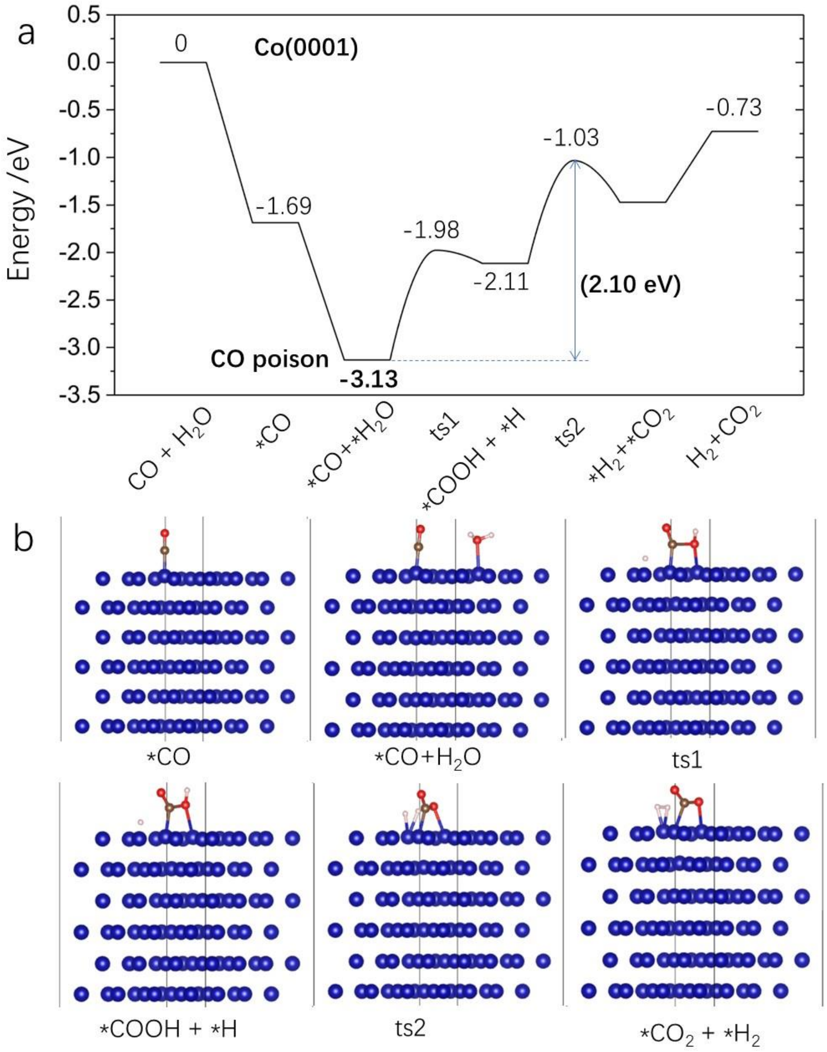

Supplementary Fig. 34. Reaction pathway for the water gas shift reaction over Co(0001). The *x*-axis shows the reaction intermediates and transition states; the *y*-axis demonstrates the relative energy.

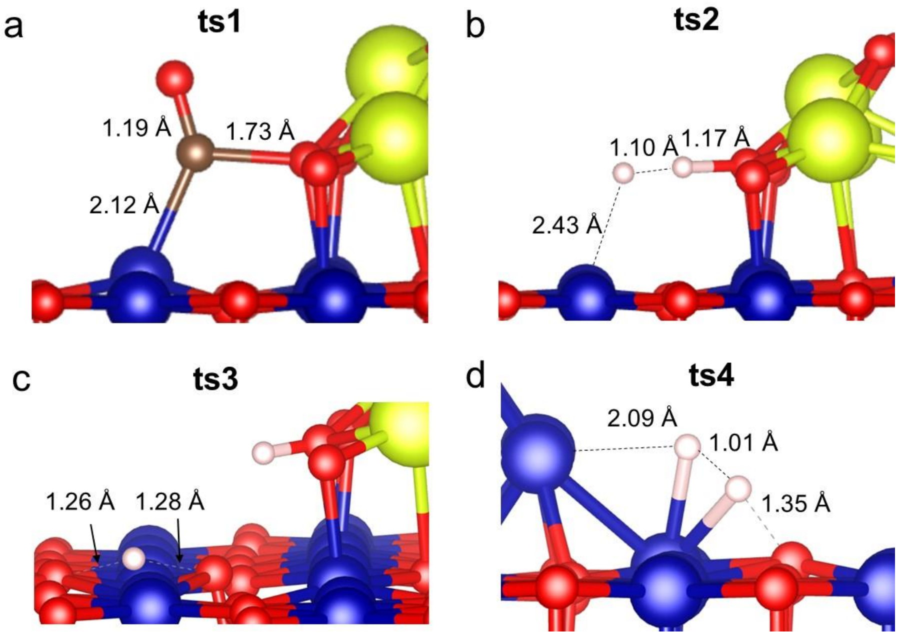

Supplementary Fig. 35. Detailed illustration of the transition state involved in Figure 6.

Supplementary Table 1. Comparison of catalytic performances for the WGS reaction over various catalysts.

Catalysts	Tem. (°C)	Gas Compositions ($mL\cdot g^{-1}\cdot h^{-1}$)	Conv. (%)	Reaction Rate ($mmol_{CO}\cdot g_{cat}^{-1}\cdot h^{-1}$)	Ref.
$1Ce9CoO_x$	250	2%CO/10%H_2O/Ar (840, 000)	25.0	169.3	This work
	250	2%CO/10%H_2O/Ar (168, 000)	63.0	85.4	
$9Ce1CoO_x$	250	2%CO/10%H_2O/Ar (168, 000)	0.3	0.4	
$1CeO_2+9CoO_x$[a]	250	2%CO/10%H_2O/Ar (168, 000)	5.2	7.2	
$1Al9CoO_x$	250	2%CO/10%H_2O/Ar (168, 000)	37.0	50.1	
Co_3O_4	250	2%CO/10%H_2O/Ar (168, 000)	7.3	9.9	
CoO-CeO_2	250	2%CO/10%H_2O/Ar (168, 000)	18.0	24.4	
Co_3O_4-CeO_2	250	2%CO/10%H_2O/Ar (168, 000)	1.0	1.4	
Co_2C	240	3%CO/26.1H_2O/29.9%H_2/N_2 (–)	44.8	10.4	13
K/Co_2C	240	3%CO/26.1H_2O/29.9%H_2/N_2 (–)	97	21.6	
Co-800N/CeO_2	260	2%CO/10%H_2O/N_2 (84, 000)	~10%	6.8	14
1Au_Cluster	270	2%CO/10%H_2O/Ar (42, 000)	82	27.8	15
1Au_Particle	270	2%CO/10%H_2O/Ar (42, 000)	20	6.8	
CeO_2/Cu	250	2%CO/10%H_2O/Ar (42, 000)	65	22.0	16
Cu/CeO_2	250	2%CO/10%H_2O/Ar (42, 000)	13	4.4	
CuZnAl	250	2%CO/10%H_2O/Ar (42, 000)	–	64.8	

[a] The catalysts were physically mixed with the same Ce/Co ratio of $1Ce9CoO_x$.

Supplementary Table 2. The components information of Co-based catalysts discussed in this work.

Catalysts	Components[a]
1Ce9CoO$_x$-fresh	CeO_2, Co_3O_4
1Ce9CoO$_x$-used	CeO_{2-x}, CoO_{1-x}, Co
CoO-CeO_2	CeO_2, CoO
Co_3O_4-fresh	Co_3O_4
Co_3O_4-used	Co
1Ce9CoO$_x$-200-fresh[b]	CeO_2, Co_3O_4
1Ce9CoO$_x$-320-fresh[b]	CeO_2, CoO
1Ce9CoO$_x$-350-fresh[b]	CeO_2, CoO, Co
1CeO_2+9CoO$_x$-fresh[c]	CeO_2, Co_3O_4
1CeO_2+9CoO$_x$-used[c]	CeO_2, Co
CeO_2-fresh	CeO_2
CeO_2-used	CeO_{2-x}

[a] Determined by XRD, XPS and Raman analysis.
[b] 1Ce9CoO$_x$ sample pretreated by 5%H_2/Ar at various temperatures for 1h.
[c] Physically mixing CeO_2 nanoparticles and Co_3O_4 sample with Ce/Co atomic ratio of 1/9.

Supplementary Table 3. EXAFS fitting results (*R*: distance; CN: coordination number) of the used $1Ce9CoO_x$ catalysts.

Sample	Co–O		Co-Co		σ^2 (Å^2)	ΔE_0
	R (Å)	CN	*R* (Å)	CN		eV
			2.50±0.01	6.9±0.4		
**$1Ce9CoO_x$-used**	1.90±0.01	3.4±0.2	2.81±0.01	4.3±0.6	0.006 (O)<br>0.009 (Co)	-2.2±1.7
			3.37±0.02	3.6±0.7		
**Co foil**	–	–	2.484±0.003	12	0.007 (Ru)	7.3±0.5

Reference:

1 Qi, L. *et al.* Influence of cerium precursors on the structure and reducibility of mesoporous CuO-CeO_2 catalysts for CO oxidation. *Appl. Catal. B: Environ.* **119-120**, 308-320, doi:10.1016/j.apcatb.2012.02.029 (2012).

2 Rodriguez, J. A. *et al.* Water-gas shift reaction on a highly active inverse CeO_x/Cu111 catalyst: unique role of ceria nanoparticles. *Angew. Chem. Int. Ed.* **48**, 8047-8050, doi:10.1002/anie.200903918 (2009).

3 Kresse, G. & Joubert, D. J. P. r. b. From ultrasoft pseudopotentials to the projector augmented-wave method. **59**, 1758 (1999).

4 Kresse, G. & Furthmüller, J. J. C. m. s. Efficiency of ab-initio total energy calculations for metals and semiconductors using a plane-wave basis set. **6**, 15-50 (1996).

5 Kresse, G. & Furthmüller, J. J. P. r. B. Efficient iterative schemes for ab initio total-energy calculations using a plane-wave basis set. **54**, 11169 (1996).

6 Tang, Y., Zhao, S., Long, B., Liu, J.-C. & Li, J. J. T. J. o. P. C. C. On the nature of support effects of metal dioxides MO_2 (M= Ti, Zr, Hf, Ce, Th) in single-atom gold catalysts: importance of quantum primogenic effect. **120**, 17514-17526 (2016).

7 Perdew, J. P., Burke, K. & Ernzerhof, M. J. P. r. l. Generalized gradient approximation made simple. **77**, 3865 (1996).

8 Henkelman, G., Uberuaga, B. P. & Jónsson, H. J. T. J. o. c. p. A climbing image nudged elastic band method for finding saddle points and minimum energy paths. **113**, 9901-9904 (2000).

9 Lykhach, Y. *et al.* Water Chemistry on Model Ceria and Pt/Ceria Catalysts. *J. Phys. Chem. C* **116**, 12103-12113, doi:10.1021/jp302229x (2012).

10 Bosio, N., Schaefer, A. & Gronbeck, H. Can oxygen vacancies in ceria surfaces be measured by O1s photoemission spectroscopy? *J Phys Condens Matter* **34**, doi:10.1088/1361-648X/ac4f7b (2022).

11 Tian, Y. *et al.* Engineering Crystallinity and Oxygen Vacancies of Co(II) Oxide Nanosheets for High Performance and Robust Rechargeable Zn–Air Batteries. *Adv. Funct. Mater.* **31**, doi:10.1002/adfm.202101239 (2021).

12 Sun, Z. *et al.* Deterministic Role of Concentration Surplus of Cation Vacancy over Anion Vacancy in Bipolar Memristive NiO. *ACS. Appl. Mater. Interfaces* **8**, 11583-11591, doi:10.1021/acsami.6b01400 (2016).

13 Gnanamani, M. K. *et al.* Low temperature water–gas shift reaction over alkali metal promoted cobalt carbide catalysts. *Top. Catal.* **57**, 612-618, doi:10.1007/s11244-013-0219-7 (2013).

14 Sun, X.-C. *et al.* Weakening the metal–support interactions of M/CeO_2 (M = Co, Fe, Ni) using a NH_3-treated CeO_2 Support for an enhanced water–gas shift reaction. *ACS Catal.* **12**, 11942-11954, doi:10.1021/acscatal.2c03664 (2022).

15 Fu, X.-P. *et al.* Direct identification of active surface species for the water–gas shift reaction on a gold–ceria catalyst. *J. Am. Chem. Soc.* **141**, 4613-4623 (2019).

16 Yan, H. *et al.* Construction of stabilized bulk-nano interfaces for highly promoted inverse CeO2/Cu catalyst. *Nat. Commun.* **10**, 3470, doi:10.1038/s41467-019-11407-2 (2019).
